# Range extension of *Odorrana nagao* (Anura, Ranidae) to south-western China, with comments on the limestone-associated *Odorrana lipuensis* species group

**DOI:** 10.3897/BDJ.14.e213369

**Published:** 2026-10-14

**Authors:** Han-Ming Song, Dan-Yang Zhou, Shuo Qi, Ming-Xing Lu, Nikolay A. Poyarkov, Tan Van Nguyen, Ying-Yong Wang

**Affiliations:** 1 School of Ecology, Sun Yat-sen University, Shenzhen 518107, Guangdong, China School of Ecology, Sun Yat-sen University, Shenzhen 518107 Guangdong China; 2 School of Life Sciences, Sun Yat-sen University, Guangzhou 510275, Guangdong, China School of Life Sciences, Sun Yat-sen University, Guangzhou 510275 Guangdong China; 3 Administration of Malipo Laoshan Provincial Nature Reserve, Wenshan 663604, Yunnan, China Administration of Malipo Laoshan Provincial Nature Reserve, Wenshan 663604 Yunnan China; 4 Department of Vertebrate Zoology, Lomonosov Moscow State University, Leninskiye Gory, GSP–1, Moscow, Russia Department of Vertebrate Zoology, Lomonosov Moscow State University, Leninskiye Gory, GSP–1 Moscow Russia; 5 The School of Medicine and Pharmacy, Duy Tan University, Da Nang, Vietnam The School of Medicine and Pharmacy, Duy Tan University Da Nang Vietnam; 6 Department of Biological Sciences, Graduate School of Science, Tokyo Metropolitan University, Hachioji, Tokyo, Japan Department of Biological Sciences, Graduate School of Science, Tokyo Metropolitan University, Hachioji Tokyo Japan

**Keywords:** China–Vietnam border, limestone karst, Malipo County, molecular phylogeny, range extension

## Abstract

**Background:**

*Odorrana nagao*, a member of the predominantly limestone-associated *O. lipuensis* species group, was recently described from limestone karst forest in Tuyen Quang Province, northern Vietnam and was previously known only from its type locality. During fieldwork in Laoshan Provincial Nature Reserve, Malipo County, Yunnan Province, China, three adult female *Odorrana*, referable to this species group, were collected. We examined their taxonomic identity using mitochondrial DNA sequence data and morphological comparisons.

**New information:**

We document the occurrence of *Odorrana nagao* in China and the presence of the *O. lipuensis* species group in Yunnan Province. Molecular phylogenetic analyses placed the Chinese specimens with the type series of *O. nagao* with strong support, while their identification was further supported by low mitochondrial genetic divergence and agreement in external morphology. The newly-examined specimens extend the known female snout–vent length from 47.1–54.7 mm to 47.1–59.5 mm. This record extends the known distribution of *O. nagao* northwards across the China–Vietnam border, increases the recognised *Odorrana* fauna of China from 47 to 48 species and brings the number of species recorded from Yunnan to 17.

## Introduction

The genus *Odorrana* Fei, Ye & Huang, comprises Asian cascade frogs distributed from north-eastern India and southern mainland China through mainland Southeast Asia to the Malay Peninsula and Greater Sunda Islands and eastwards to Hainan, Taiwan islands and the Ryukyu Archipelago (Frost 2026). Most species inhabit forested streams, although several congeners are closely associated with limestone landscapes, including karst streams, cave entrances and subterranean aquatic habitats (Song et al. 2023, Song et al. 2025,Nguyen et al. 2026a, Nguyen et al. 2026b, Pham et al. 2026). The fragmented distribution and environmental heterogeneity of karst systems may restrict dispersal and promote local differentiation, while the limited accessibility of many limestone areas means that their amphibian diversity remains incompletely documented (Clements et al. 2006, Lin et al. 2022, Song et al. 2025).

China supports particularly high diversity of *Odorrana*. Before the present study, 47 species were recorded from the country, including 32 species considered endemic to China (AmphibiaChina 2026, Frost 2026). Knowledge of this fauna has increased rapidly through descriptions of new species, taxonomic revisions and range extensions, particularly in southern China and along its border with northern Vietnam. The *O. lipuensis* species group represents a phylogenetically distinct and predominantly limestone-associated lineage currently comprising *Odorrana calciphila* Song, Qi, Wang, Liu & Wang, *O. concelata* Wang, Zeng & Lin, *O. feii* Li, Mu, Jing, Liu, Cheng & Wang, *O. liboensis* Luo, Wang, Xiao, Wang & Zhou, *O. lipuensis* Mo, Chen, Wu, Zhang & Zhou and *O. nagao* Pham, Hoang, Nguyen, Nguyen, Ngo, Le & Ziegler (Song et al. 2025, Li et al. 2025, Pham et al. 2026). Members of the group are morphologically characterised by the following combination of features: adult females generally larger than males; external vocal sacs absent in males; discs of Fingers III and IV prominently enlarged and transversely oval, with disc of Finger IV widest; tip of Finger I lacking a lateroventral groove; dorsal skin relatively smooth, with minute flattened granules forming a fine vermiculate texture; and dorsum typically moss-green with irregular brown mottling (Lin et al. 2022, Li et al. 2025, Song et al. 2025, Pham et al. 2026). Members of the group are currently known mainly from limestone regions of Guangdong, Guangxi and Guizhou in southern China and northern Vietnam. The recognised diversity of the group has increased markedly over the past decade, with all six currently recognised species having been described since 2015. These recent discoveries indicate that both the diversity and geographic distribution of this lineage remain incompletely understood, particularly in the extensive, but insufficiently surveyed karst landscapes of southern China and northern Vietnam (e.g.Mo et al. 2015, Pham et al. 2016, Pham et al. 2026, Song et al. 2023

*Odorrana nagao* was recently described by Pham et al. (2026) from Cham Chu Nature Reserve, Tuyen Quang Province, northern Vietnam, based on morphological and molecular evidence. The species can be distinguished from its congeners by the following combination of characters: body of moderate size, with SVL 37.4 mm in the known male and 47.1–54.7 mm in females of the type series; head longer than wide; tympanum distinct, round and approximately half the eye diameter; vomerine teeth present; male lacking an external vocal sac, but bearing a nuptial pad on Finger I; toe webbing formula I0–1II0–1III0–1⅓IV1⅓–¼V; tibio-tarsal articulation reaching the tip of the snout when the hind-limb is adpressed; dorsolateral conical spines and a dorsolateral fold present; dorsum moss-green with brown mottling; and ventral surfaces cream with dark-brown mottling (Pham et al. 2026). Before the present study, the species was known only from its type locality.

During fieldwork in May 2026, three adult female *Odorrana*, referable to the *O. lipuensis* species group, were collected in Laoshan Provincial Nature Reserve, Malipo County, south-eastern Yunnan, close to the international border with Vietnam. We examined these specimens using morphological comparisons and mitochondrial DNA sequence data. Herein, we document the occurrence of *O. nagao* in China, update its known distribution and discuss the implications of this finding for the diversity, distribution and biogeography of the *O. lipuensis* species group.

## Materials and methods

### Sampling

**Figure 1.**
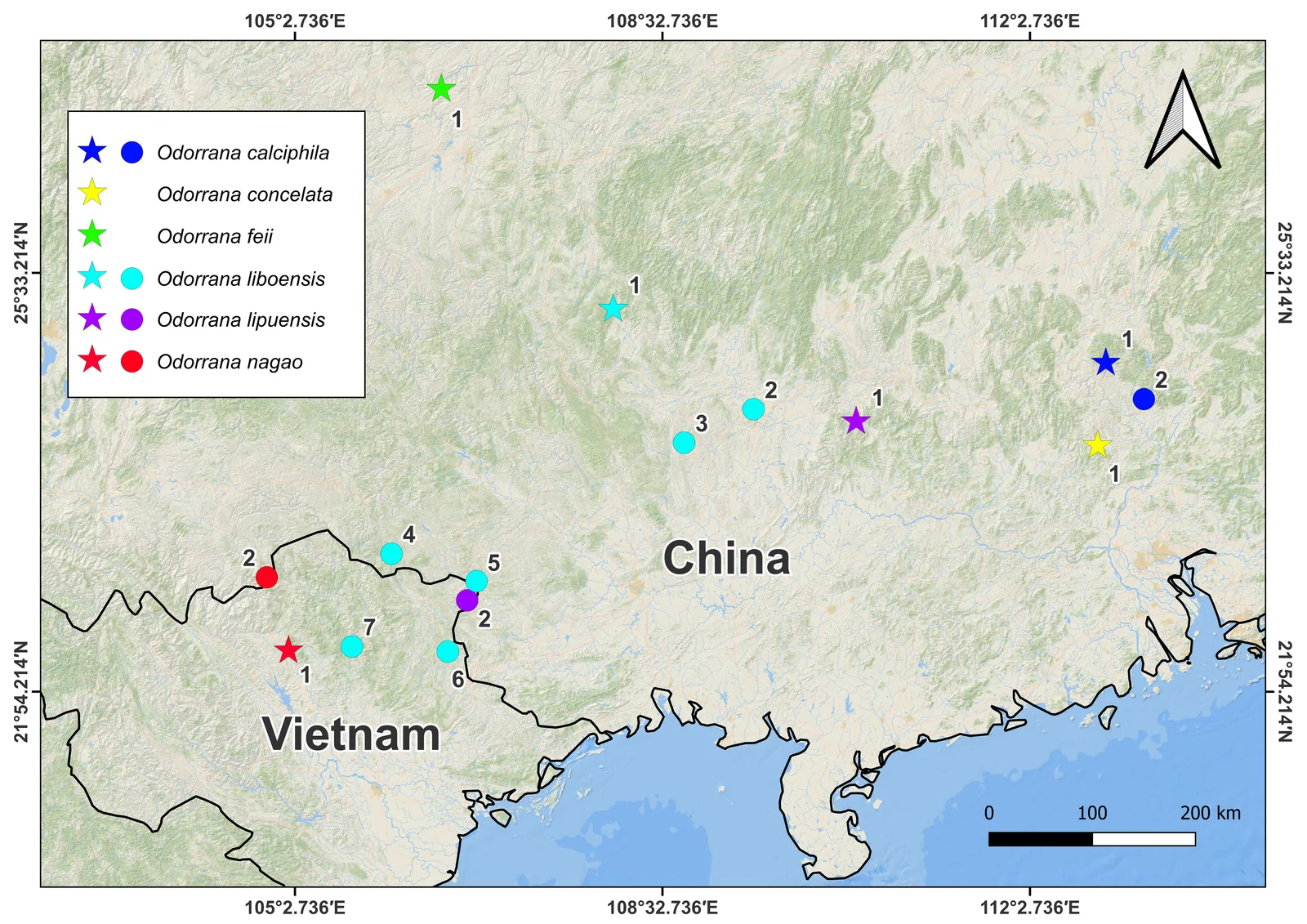
Distribution of species of the *Odorrana lipuensis* group in southern China and northern Vietnam. Stars indicate type localities and circles represent additional records; numbers correspond to the localities listed in Suppl. material 1.

Three specimens of *Odorrana* were collected during fieldwork in Malipo County, Yunnan Province, China (Fig. 1). The specimens were euthanised using tricaine methanesulphonate (MS-222), fixed in 10% buffered formalin and subsequently transferred to 75% ethanol for permanent preservation in the herpetological collection of Sun Yat-sen University (SYS), Guangzhou, Guangdong, China. Thigh-muscle tissue was removed before fixation and preserved in 95% ethanol for subsequent molecular analyses. Geographic coordinates were recorded using the WGS 84 datum and elevation was measured at the collection locality.

### Laboratory protocols

Genomic DNA was extracted from tissue samples using a DNA extraction kit (Tiangen Biotech Co., Ltd, Beijing, China), following the manufacturer’s protocol. Partial fragments of the mitochondrial 16S ribosomal RNA gene (16S) and cytochrome *c* oxidase subunit I gene (COI) were amplified by polymerase chain reaction using the primers and conditions provided by Lyu et al. (2019). PCR products were purified using spin columns and sequenced with the forward primers and BigDye Terminator Cycle Sequencing Kit (Applied Biosystems, Waltham, MA, USA). Sequencing was performed by Wuhan Tianyi Huiyuan Bioscience and Technology Inc. using an ABI Prism 3730 automated DNA sequencer. Newly-generated sequences were deposited in GenBank under the accession numbers provided in Table 1.

### Molecular phylogenetic analyses

The molecular dataset comprised 25 samples, including 23 ingroup samples of *Odorrana* and two samples of *Nidirana* Dubois, 1992 used as outgroups (Table 1). Sequences were either generated in the present study or downloaded from GenBank. Newly-generated sequences were assembled and manually checked in SeqMan Pro 7.1.0 (DNASTAR Inc., Madison, WI, USA). Sequences were aligned using MAFFT v.7.037b under default settings (Katoh et al. 2005), manually inspected and concatenated in MEGA 11 (Tamura et al. 2021). COI sequences were translated into amino-acid sequences to verify the reading frame and check for premature stop codons.

The best-fitting nucleotide-substitution models were selected separately for 16S and the three COI codon positions in IQ-TREE 3.1.1 using ModelFinder and the Bayesian information criterion (Chernomor et al. 2016, Kalyaanamoorthy et al. 2017, Wong et al. 2026). The selected models were TIM2+F+G4 for 16S, HKY+I for the first and second COI codon positions and TN+F+G4 for the third COI codon position.

Phylogenetic relationships were inferred using Maximum Mikelihood (ML) and Bayesian Inference (BI). The ML analysis was performed in IQ-TREE 3.1.1, with nodal support assessed using 1,000 SH-like approximate likelihood-ratio test replicates and 1,000 ultrafast bootstrap replicates (Hoang et al. 2018). The BI analysis was conducted in MrBayes 3.2.7a (Ronquist et al. 2012) using two independent runs, each comprising one cold and three heated Markov chains. The analyses were run for 100 million generations, sampled every 1,000 generations, with the first 25% of samples discarded as burn-in. Convergence was accepted when the average standard deviation of split frequencies was below 0.005. Nodes with Bayesian posterior probabilities ≥ 0.90, SH-aLRT values ≥ 80 and ultrafast bootstrap values ≥ 95 were considered strongly supported.

### Genetic distances

Pairwise Kimura two-parameter distances within the *O. lipuensis* species group were calculated separately for 16S and COI in MEGA 11 using the pairwise-deletion option (Kimura 1980).

### Morphological examination

The three specimens were measured to the nearest 0.1 mm on the left side of the body using digital callipers (Deli DL91200). Measurements and abbreviations followed Fei and Ye (2016) and Jiang and Li (2021)): snout–vent length (SVL), from tip of snout to posterior margin of vent; head length (HDL), from tip of snout to articulation of head and trunk; head width (HDW), maximum width of head; snout length (SNT), from tip of snout to anterior corner of eye; internarial distance (IND), distance between nares; interorbital distance (IOD), minimum distance between upper eyelids; eye diameter (ED), distance between anterior and posterior corners of eye; tympanum diameter (TD), maximum diameter of tympanum; hand length (HND), from wrist to tip of Finger III; radio-ulna length (RAD), from elbow to wrist; foot length (FTL), from heel to tip of Toe IV; and tibia length (TIB), from knee to heel. External morphology was examined with the naked eye and a Leica EZ4 HD stereomicroscope. Sex was determined by examination of the gonads following dissection. Morphological terminology followed Zhou (1956) and Fei and Ye (2016) and comparative data for *O. nagao* were obtained from its original description (Pham et al. 2026).

## Taxon treatments

### Odorrana nagao

Pham, Hoang, Nguyen, Nguyen, Ngo, Le & Ziegler, 2026

A45ADCF8-9A02-5FDF-9983-9E2F3E5F0A9A

#### Materials

- **Type status:** Other material. **Occurrence:** catalogNumber: SYS a009830; recordedBy: Han-Ming Song; individualCount: 1; sex: female; lifeStage: adult; occurrenceStatus: present; occurrenceID: 802A115D-D2B0-5A1B-86D6-D3C4918BA087; **Taxon:** scientificName: *Odorrana nagao*; order: Anura; family: Ranidae; genus: *Odorrana*; specificEpithet: *nagao*; vernacularName: Nagao Odorous Frog; **Location:** country: China; countryCode: CN; stateProvince: Yunnan Province; county: Malipo County; locality: Laoshan Provincial Nature Reserve; verbatimElevation: 1,030 m a.s.l.; verbatimCoordinates: 23.0141°N, 104.8283°E; **Event:** eventDate: 15-05-2026; **Record Level:** language: en; basisOfRecord: PreservedSpecimen; **Material Entity:** disposition: in collection
- **Type status:** Other material. **Occurrence:** catalogNumber: SYS a009831; recordedBy: Han-Ming Song; individualCount: 1; sex: female; lifeStage: adult; occurrenceStatus: present; occurrenceID: CB97B728-016C-5437-B2B7-C9FA741AAEB7; **Taxon:** scientificName: *Odorrana nagao*; order: Anura; family: Ranidae; genus: *Odorrana*; specificEpithet: *nagao*; vernacularName: Nagao Odorous Frog; **Location:** country: China; countryCode: CN; stateProvince: Yunnan Province; county: Malipo County; locality: Laoshan Provincial Nature Reserve; verbatimElevation: 1,030 m a.s.l.; verbatimCoordinates: 23.0141°N, 104.8283°E; **Event:** eventDate: 15-05-2026; **Record Level:** language: en; basisOfRecord: PreservedSpecimen; **Material Entity:** disposition: in collection
- **Type status:** Other material. **Occurrence:** catalogNumber: SYS a009832; recordedBy: Han-Ming Song; individualCount: 1; sex: female; lifeStage: adult; occurrenceStatus: present; occurrenceID: 4A981881-BD0F-53D4-9EEA-1DF3AB75B7BD; **Taxon:** scientificNameID: *Odorrana nagao*; order: Anura; family: Ranidae; genus: *Odorrana*; specificEpithet: *nagao*; vernacularName: Nagao Odorous Frog; **Location:** country: China; countryCode: CN; stateProvince: Yunnan Province; county: Malipo County; locality: Laoshan Provincial Nature Reserve; verbatimElevation: 1,030 m a.s.l.; verbatimCoordinates: 23.0141°N, 104.8283°E; **Event:** eventDate: 15-05-2026; **Record Level:** language: en; basisOfRecord: PreservedSpecimen; **Material Entity:** disposition: in collection

#### Description

**Suggested Chinese name**. 宣光臭蛙 (xuān guāng chòu wā)

**Description of the specimens from China (n = 3)**

Measurements of the specimens are presented in Table 2. All three specimens are adult females of moderate size (SVL 56.6–59.5 mm). Body relatively slender. Head longer than wide (HDW/HDL 0.92–0.93), its length approximately 36–38% of SVL. Snout obtusely rounded in dorsal view, projecting beyond lower jaw and rounded in profile; snout longer than eye diameter (SNT/ED 1.08–1.30). Canthus rostralis distinct; loreal region slightly concave and oblique. Nostrils lateral, closer to tip of snout than to eye. Internarial distance slightly greater than interorbital distance (IND/IOD 1.04–1.12). Pupil horizontally oval. Tympanum distinct and round, smaller than eye (TD/ED 0.62–0.65). Supratympanic fold weakly developed, extending posterodorsally from posterior corner of eye, passing above dorsal margin of tympanum and terminating near shoulder. Tiny spinules present along anterior and posterior margins of tympanum. Vomerine teeth present on two oblique ridges; tongue cordiform and deeply notched posteriorly.

Fore-limbs slender; radio-ulna length approximately 21–22% of SVL; hand length approximately 29–32% of SVL. Fingers slender, relative lengths II < I < IV < III. Tips of Fingers II–IV expanded into discs with lateroventral grooves; discs of Fingers III and IV prominently enlarged and transversely oval, disc of Finger IV widest; tip of Finger I lacking lateroventral grooves. Fingers free of webbing. Subarticular tubercles round, formula 1, 1, 2, 2; inner metacarpal tubercle elongate; outer metacarpal tubercle small. Nuptial pads absent.

Hind-limbs long and slender; tibia length approximately 53–57% of SVL; foot length approximately 72–77% of SVL. Tibio-tarsal articulation reaching tip of snout when hind-limb adpressed along body. Toes slender, relative lengths I < II < III < V < IV; tips expanded into discs with lateroventral grooves. Toe webbing well developed, formula I0–1II0–1III0–1⅓IV1⅓–¼V. Subarticular tubercles present, formula 1, 1, 2, 3, 2; inner metatarsal tubercle oval; outer metatarsal tubercle absent.

Dorsal surfaces of head and body relatively smooth, with dense minute flattened granules forming a fine vermiculate texture. Small tubercles present on flanks and dorsal surfaces of fore- and hind-limbs. Dorsolateral fold distinct; conical spines present along dorsolateral region. Throat, chest, belly and ventral surfaces of thighs smooth.

**Figure 2.**
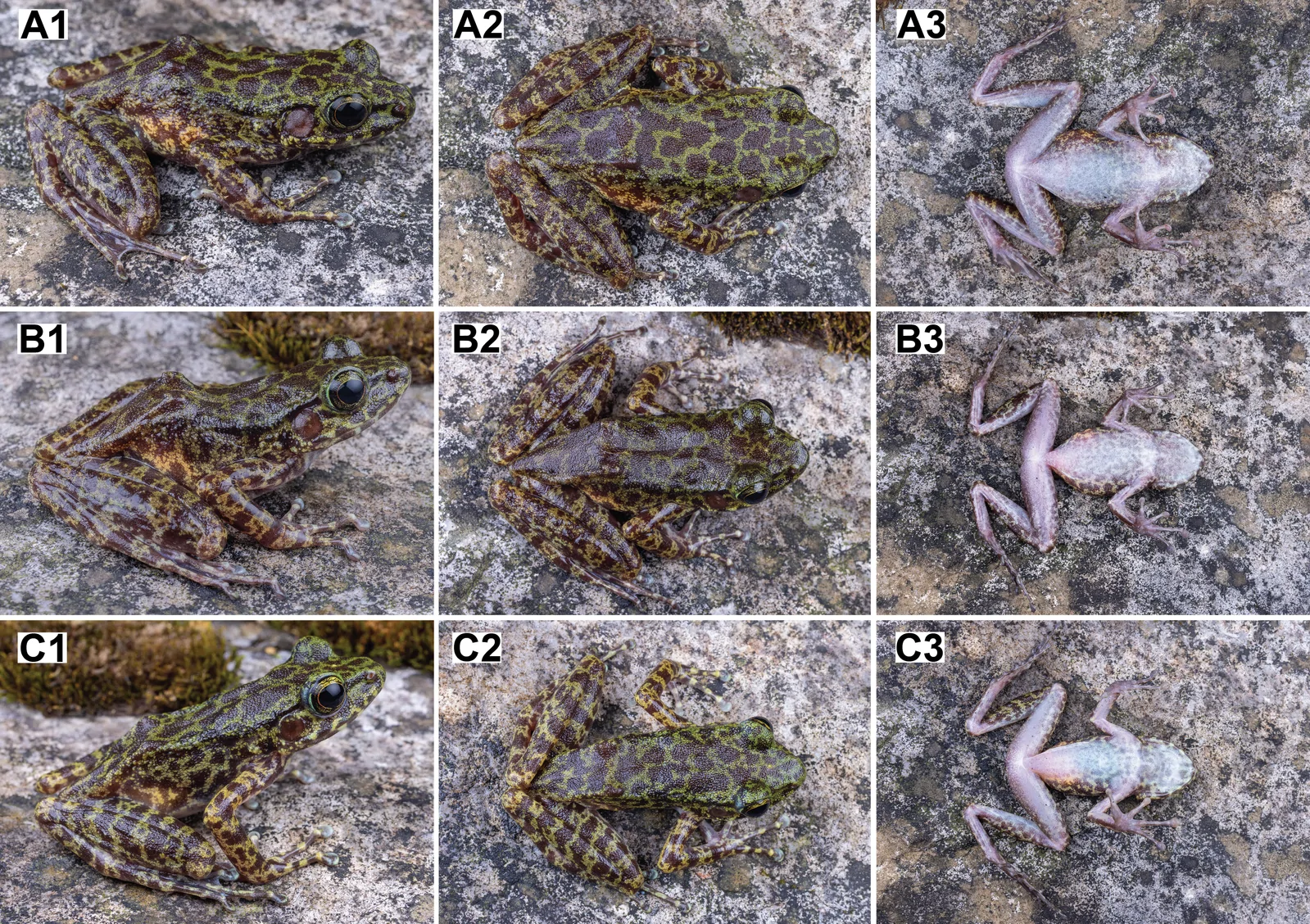
Colouration in life of *Odorrana nagao* from Malipo, Yunnan, China: lateral (A1, B1, C1), dorsal (A2, B2, C2) and ventral (A3, B3, C3) views of SYS a009830 (A1–A3), SYS a009831 (B1–B3) and SYS a009832 (C1–C3), respectively. Photographs by Dan-Yang Zhou.

**Colouration in life (Fig. 2)**. Dorsal surfaces moss-green to olive-green, with irregular dark-brown blotches forming a reticulate pattern on head and body. Flanks brownish-green with darker mottling. Upper surfaces of limbs greenish-brown, bearing distinct dark-brown transverse bars. Upper lip mottled brown and cream; iris golden brown. Ventral surfaces creamy-white; throat and chest suffused with pale greyish-brown, abdomen largely immaculate; ventral surfaces of limbs pale flesh-grey. Palmar and plantar surfaces greyish-brown.

**Figure 3.**
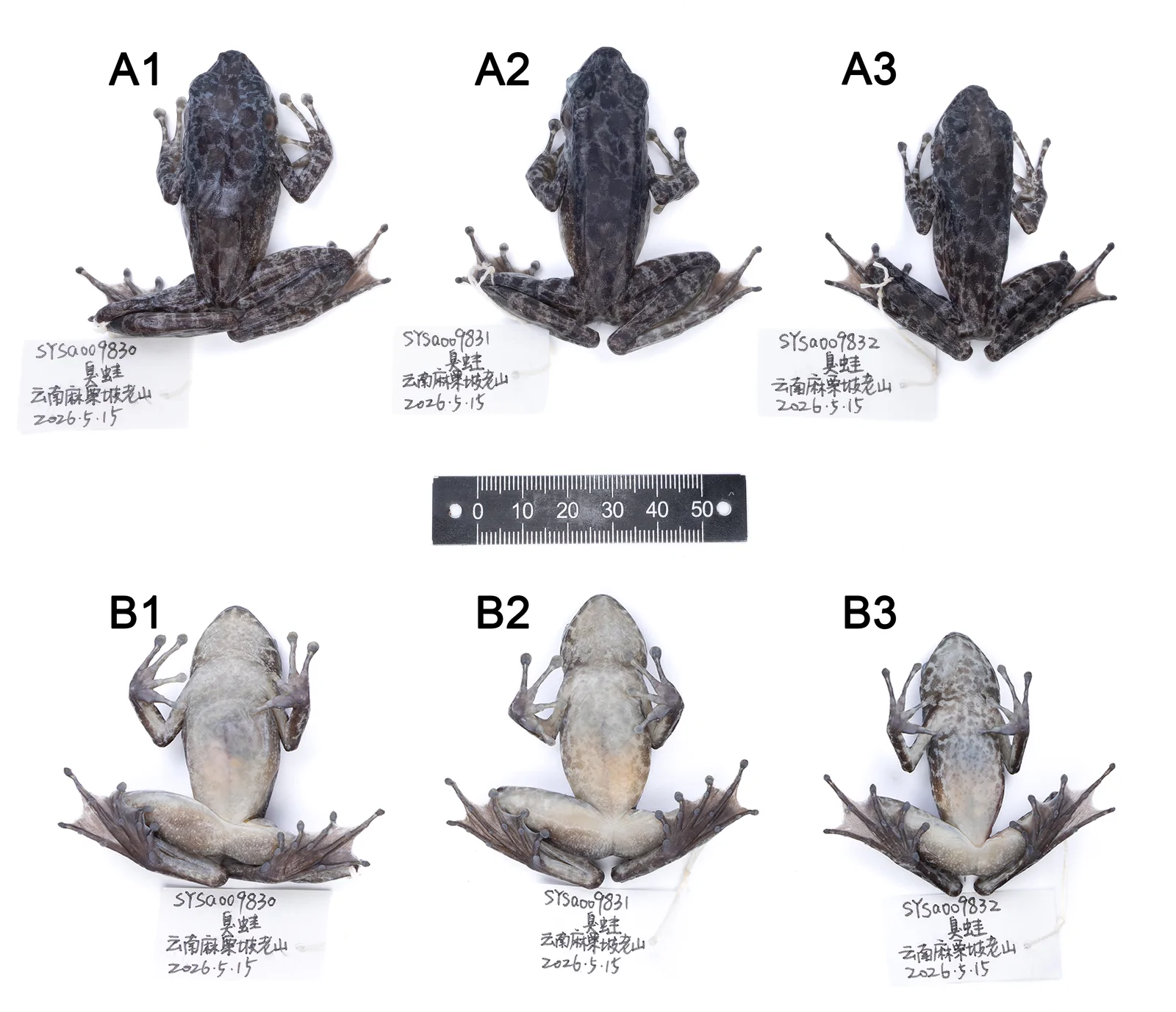
Preserved specimens of *Odorrana nagao* from Malipo, Yunnan, China: dorsal (A1–A3) and ventral (B1–B3) views of SYS a009830, SYS a009831 and SYS a009832, respectively. Photographs by Dan-Yang Zhou.

**Colouration in preservative (Fig. 3)**. After preservation, green colouration faded completely. Dorsal surfaces became dark greyish-brown, with darker blotches and pale-grey marbling remaining visible; transverse bars on limbs remained distinct. Ventral surfaces became cream to pale grey, with diffuse greyish-brown mottling on throat, chest and abdomen, more pronounced in SYS a009832. Palmar and plantar surfaces dark greyish-brown; webbing pale grey.

**Variation**. The three Chinese specimens generally agree with one another and with the original description of *O. nagao* in external morphology. Minor variation occurs in body size, morphometric proportions and colour pattern. SYS a009831 is the largest specimen (SVL 59.5 mm), whereas SYS a009830 and SYS a009832 measure 56.6 and 57.0 mm, respectively. Relative tympanum diameter is nearly invariant amongst the three specimens (TD/ED 0.62–0.65). The internarial distance is slightly greater than the interorbital distance in the Chinese specimens (IND/IOD 1.04–1.12), whereas the opposite condition was reported for the male holotype (IND/IOD 0.94). This difference is considered intraspecific variation. The Chinese specimens are also larger than females of the type series (SVL 56.6–59.5 mm versus 47.1–54.7 mm), extending the known female SVL range of the species to 47.1–59.5 mm. Colour pattern is variable. Dorsal brown markings range from irregular mottling to more clearly defined rounded blotches. Ventral mottling varies from distinct dark-grey or dark-brown markings on throat, chest and lateral belly to an almost immaculate creamy-white condition. No diagnostically relevant morphological differences were observed between the Chinese specimens and the type series.

**Revised diagnosis**. Based on the original description by Pham et al. (2026) and the additional specimens examined in the present study, *Odorrana nagao* is distinguishable from its congeners by the following combination of characters: (1) body medium-sized, SVL 37.4 mm in the known male and 47.1–59.5 mm in females; (2) head longer than wide; (3) tympanum distinct, round, approximately one-half to two-thirds of eye diameter; (4) vomerine teeth present; (5) external vocal sac absent in male; (6) nuptial pad present on finger I in male; (7) toe webbing formula I0–1II0–1III0–1⅓IV1⅓–¼V; (8) tibio-tarsal articulation reaching the tip of the snout when the hind-limb is adpressed along the body; (9) dorsolateral conical spines present; (10) dorsolateral folds present; (11) dorsum moss-green, with brown markings varying from irregular mottling to rounded blotches; and (12) ventral surfaces cream to creamy-white, with variable dark-grey or dark-brown mottling on the throat, chest and lateral belly or nearly immaculate.

#### Distribution

*Odorrana nagao* was previously known only from its type locality in Cham Chu Nature Reserve, Tuyen Quang Province, northern Vietnam (Pham et al. 2026). The Chinese locality lies approximately 75 km north of the type locality and considerably extends the known distribution of the species (Fig. 1). Both confirmed localities occur within protected areas in the transboundary limestone belt of northern Vietnam and southern China. The presence of extensive karst landscapes between and around these localities suggests that the species may be more widely distributed than currently documented.

#### Ecology

In China, *O. nagao* was recorded in subtropical broadleaved forest on limestone karst at an elevation of 1,030 m a.s.l. This record extends the known elevational range of the species from 641–773 m at the type locality in northern Vietnam to 641–1,030 m a.s.l., raising the documented upper elevational limit by 257 m. All three specimens were encountered at night, after complete darkness, on limestone rocks near the entrance of a cave, closely resembling the microhabitat reported at the type locality. Mature ovarian oocytes were present in the adult females collected in May, suggesting reproductive activity during this period. Further surveys are required to clarify the species’ distribution, elevational range, breeding season, reproductive microhabitat and larval ecology, particularly in limestone areas between the two confirmed localities and elsewhere in south-eastern Yunnan and northern Vietnam.

#### Conservation

*Odorrana nagao* is currently known from only two confirmed localities: Cham Chu Nature Reserve in northern Vietnam and Laoshan Provincial Nature Reserve in south-eastern Yunnan, China. The occurrence of both known populations within protected areas is encouraging and may afford some protection to the species and its habitat. However, presence within protected-area boundaries alone does not demonstrate stable populations or the absence of threats. Information remains unavailable on population size, population trends, reproductive success, connectivity between the two known populations and the species’ actual geographic range. Its extent of occurrence and area of occupancy cannot yet be estimated reliably. Potential threats to limestone ecosystems, including quarrying, forest degradation, tourism development and hydrological alteration, have also not been evaluated across its possible range (T.V. Nguyen, pers. obs). We, therefore, provisionally recommend that *O. nagao* be assessed as Data Deficient (DD), following the IUCN Red List Categories and Criteria (IUCN Standards and Petitions Committee 2024). Targeted surveys are needed to clarify its distribution, population status, habitat requirements and exposure to threats, particularly in limestone landscapes between and surrounding the two confirmed localities.

## Analysis

**Phylogenetic relationships and genetic distances**

**Figure 4.**
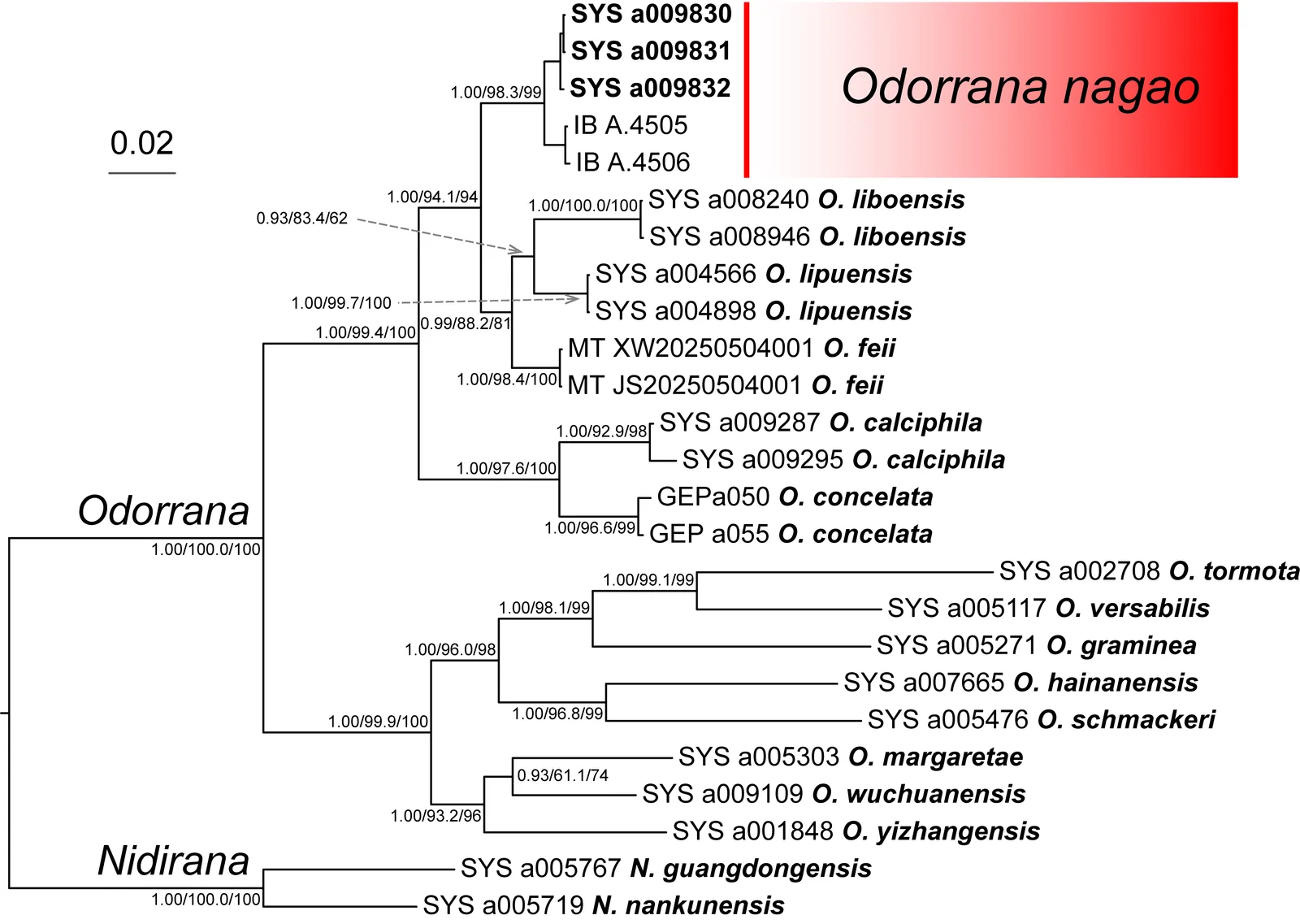
Bayesian-Inference phylogram, based on the concatenated mitochondrial 16S rRNA and COI dataset. Numbers at nodes represent Bayesian posterior probabilities (BPP), SH-like approximate likelihood-ratio test values (SH-aLRT) and ultrafast bootstrap values (UFB), respectively.

The final concatenated alignment comprised 1,630 base pairs (bp), including 1,036 bp of 16S and 594 bp of COI. Bayesian-Inference (BI) and Maximum-Likelihood (ML) analyses recovered broadly congruent topologies (Fig. 4). The six sampled species assigned to the *O. lipuensis* species group formed a well-supported lineage, distinct from the other sampled species of *Odorrana* (BPP/SH-aLRT/UFB = 1.00/99.4/100).

The three specimens from Yunnan were recovered together with two specimens from the type series of *O. nagao* from Tuyen Quang Province, Vietnam, forming a strongly supported clade (BPP/SH-aLRT/UFB = 1.00/98.3/99). Within the *O. lipuensis* species group, *O. nagao* was recovered as sister to a clade containing the remaining sampled species, with high support from the BI and SH-aLRT analyses, but slightly lower UFB support (BPP/SH-aLRT/UFB = 1.00/94.1/94).

The three Yunnan specimens exhibited very low intraspecific genetic divergence, ranging from 0 to 0.19% for 16S and their COI sequences were identical. Kimura two-parameter distances between the Yunnan specimens and the Vietnamese type series of *O. nagao* were 0.38% for 16S and 1.73–1.90% for COI. By comparison, distances between the Yunnan specimens and *O. feii*, the genetically closest sampled congener, were 2.11% for 16S and 6.25% for COI (Suppl. materials 2, 3).

The strongly supported phylogenetic placement of the Yunnan specimens with the type series, together with their low mitochondrial divergence and agreement in diagnostic external morphology, supports their identification as *O. nagao*.

## Discussion

**Range extension to China and updated species diversity**

The discovery of *O. nagao* in Laoshan Provincial Nature Reserve extends its known range approximately 75 km northwards from the type locality in Tuyen Quang Province, Vietnam. This record increases the number of *Odorrana* species naturally occurring in China from 47 to 48. Of these, 32 species are endemic to China and 17 are shared with neighbouring countries. The occurrence of *O. nagao* in south-eastern Yunnan is biogeographically plausible because Malipo County and the type locality lie within the same transboundary limestone belt. The two localities are not separated by an obvious major ecological or geographical barrier. Consequently, although the country record is new, the presence of the species in southern China is not unexpected and probably reflects incomplete survey coverage in the border region. Yunnan supports the highest documented amphibian species richness amongst the provincial-level administrative regions of China (AmphibiaChina 2026). Based on recent taxonomic and distributional updates, 17 species of *Odorrana* are currently recognised from Yunnan: *O. andersonii* (Boulenger), *O. cangyuanensis* (Yang), *O. chapaensis* (Bourret), *O. chloronota* (Günther), *O. dulongensis* Liu, Che & Yuan, *O. geminata* Bain, Stuart, Nguyen, Che & Rao, *O. grahami* (Boulenger), *O. graminea* (Boulenger), *O. heatwolei* (Stuart & Bain), *O. jingdongensis* Fei, Ye & Li, *O. junlianensis* Huang, Fei & Ye, *O. macrotympana* (Yang), *O. nagao*, *O. nasica* (Boulenger), *O. sudianensis* Kilunda, Yu, Wu & Che, *O. tiannanensis* (Yang & Li) and *O. yangi* Liu, Bu, Feng, Hou, Rao & Li (Frost 2026; this study).

**Geographic expansion of the *Odorrana lipuensis* species group**

The new record from Malipo extends the distribution of the *O. lipuensis* species group into Yunnan Province, China. The group was previously known mainly from limestone regions of Guangdong, Guangxi and Guizhou in southern China and northern Vietnam. Its recognised diversity and geographic extent have increased markedly during the past decade, with all six currently recognised species having been described since 2015. The phylogenetic analyses recovered the six sampled species as a well-supported mitochondrial lineage. Their predominantly limestone-associated distribution and occurrence across geographically separated karst blocks suggest that habitat discontinuity and restricted dispersal may have contributed to population differentiation. Nevertheless, the present dataset comprises only two mitochondrial markers and was designed primarily to establish the identity of the Yunnan specimens. It is, therefore, insufficient to resolve the deeper evolutionary history of the lineage or support changes in genus-group classification. Further work should incorporate broader taxon sampling, nuclear markers and detailed morphological comparisons. Comparative investigations of advertisement calls, osteology and larval morphology would also help determine whether members of the group possess functional specialisations, associated with limestone streams, rocky substrates and cave environments. Current evidence supports a strong ecological association with karst, but does not yet demonstrate specific morphological or functional adaptations.

**Biogeographic continuity across the China–Vietnam border**

The occurrence of *Odorrana nagao* in both northern Vietnam and southern China further highlights the close biogeographic relationship between the herpetofaunas on either side of the China–Vietnam border. This region comprises a complex mosaic of interconnected forests and limestone formations interspersed with deeply dissected mountains and river valleys, which may both facilitate faunal exchange and promote the isolation and diversification of local populations. Recent studies have repeatedly revealed comparable transboundary distributions, overlooked populations and previously unrecognised diversity, indicating that the distribution and taxonomic diversity of amphibians and reptiles along the border remain incompletely documented (e.g. Poyarkov et al. (2021), Poyarkov et al. (2023), Poyarkov et al. (2024), Vogel et al. (2024), Nguyen and Vogel (2025), Nguyen et al. (2025), Xu et al. (2025), Song et al. (2026)). The locality in Malipo lies approximately 75 km north of the type locality of *O. nagao* in Cham Chu Nature Reserve. The apparent distributional gap between the two localities is likely attributable, at least partly, to insufficient survey effort, although genuine discontinuity or population differentiation cannot yet be excluded. Extensive limestone formations and potentially suitable karst forests occur between and around the two known localities. Additional populations may, therefore, occur in Tuyen Quang and Cao Bang Provinces of northern Vietnam, Wenshan Prefecture in south-eastern Yunnan and other karst landscapes along the international border. Coordinated cross-border surveys integrating voucher specimens, morphology, molecular data and acoustic observations will be essential for refining the distribution of *O. nagao* and may reveal overlooked populations or undescribed diversity within the *O. lipuensis* species group. Such international collaboration is particularly important in this biogeographically complex and still insufficiently surveyed border region.

**Note added during peer review**

While the present manuscript was under peer review, Liu et al. (2026) published the first record of *Odorrana nagao* from China on 24 September 2026, based on a single adult female (KIZ 20211202 with SVL 55.8 mm) collected in Tianbao Town, Malipo County, Yunnan Province, on 14 June 2021. They supported its identification using morphological comparisons and mitochondrial 16S rRNA data, reporting an uncorrected genetic distance of 0.4% from the type specimens. Their publication, therefore, precedes the present account of the species in China. Our study provides independent evidence, based on three additional adult females collected in Laoshan Nature Reserve, Malipo County, on 15 May 2026, together with further morphological, molecular and ecological information. The two studies complement one another and strengthen the evidence for the occurrence of *O. nagao* in south-eastern Yunnan.

## Supplementary Material

XML Treatment for Odorrana nagao

3D909039-C6CE-5F0E-B414-3AFAAA67B5D1 10.3897/BDJ.14.e213369.suppl1 Supplementary material 1 Localities of species of the *Odorrana lipuensis* group shown in Figure 1 Data type Occurrences Brief description Locality numbers correspond to those shown for each species in Fig. 1. File: oo_1753939.xlsx https://binary.pensoft.net/file/1753939 Han-Ming Song, Dan-Yang Zhou, Shuo Qi, Ming-Xing Lu, Nikolay A. Poyarkov, Tan Van Nguyen, Ying-Yong Wang

B8C32B14-DE97-5F21-82CA-F1C7615B6506 10.3897/BDJ.14.e213369.suppl2 Supplementary material 2 Kimura two-parameter genetic distances for 16S rRNA Data type Genetic distances Brief description Pairwise Kimura two-parameter distances (%) amongst 15 samples of the *Odorrana lipuensis* group, based on 1,036 base pairs of the mitochondrial 16S rRNA gene. File: oo_1753945.xlsx https://binary.pensoft.net/file/1753945 Han-Ming Song, Dan-Yang Zhou, Shuo Qi, Ming-Xing Lu, Nikolay A. Poyarkov, Tan Van Nguyen, Ying-Yong Wang

8E8747D5-0E8D-58D4-86B0-019E58ED6EC9 10.3897/BDJ.14.e213369.suppl3 Supplementary material 3 Kimura two-parameter genetic distances for COI Data type Genetic distances Brief description Pairwise Kimura two-parameter distances (%) amongst 15 samples of the *Odorrana lipuensis* group, based on 594 base pairs of the mitochondrial COI gene. File: oo_1753957.xlsx https://binary.pensoft.net/file/1753957 Han-Ming Song, Dan-Yang Zhou, Shuo Qi, Ming-Xing Lu, Nikolay A. Poyarkov, Tan Van Nguyen, Ying-Yong Wang

## Figures and Tables

**Table 1.** Samples used in the molecular phylogenetic analyses and corresponding GenBank accession numbers.

**No**.	**Species**	**Locality**	**Specimen voucher**	**16S rRNA**	**COI**
**1**	*Odorrana nagao*	China: Yunnan: Malipo: Laoshan Provincial Nature Reserve	SYS a009830	PZ873260	PZ873263
**2**	*Odorrana nagao*	China: Yunnan: Malipo: Laoshan Provincial Nature Reserve	SYS a009831	PZ873261	PZ873264
**3**	*Odorrana nagao*	China: Yunnan: Malipo: Laoshan Provincial Nature Reserve	SYS a009832	PZ873262	PZ873265
**4**	*Odorrana nagao*	Vietnam: Tuyen Quang: Cham Chu Nature Reserve: Cao Duong Village	IB A.4505	PZ332369	PZ334602
**5**	*Odorrana nagao*	Vietnam: Tuyen Quang: Cham Chu Nature Reserve: Cao Duong Village	IB A.4506	PZ332370	PZ334603
**6**	*Odorrana calciphila*	China: Guangdong: Yangshan: Loushuiping Village	SYS a009287	PV347066	PV341280
**7**	*Odorrana calciphila*	China: Guangdong: Ruyuan: Datan River Nature Reserve	SYS a009295	PV347070	PV341284
**8**	*Odorrana concelata*	China: Guangdong: Qingyuan: Longlinchang Village	GEP a050	OP137167	–
**9**	*Odorrana concelata*	China: Guangdong: Qingyuan: Longlinchang Village	GEP a055	OP137172	–
**10**	*Odorrana feii*	China: Guizhou: Xiuwen County	MT XW20250504001	PV951782	PV943472
**11**	*Odorrana feii*	China: Guizhou: Jinsha County	MT JS20250504001	PV951785	PV943475
**12**	*Odorrana graminea*	China: Hainan: Wuzhishan: Mt. Wuzhi	SYS a005271	OR658961	OR659601
**13**	*Odorrana hainanensis*	China: Hainan: Lingshui: Mt. Diaoluo	SYS a007665	OR658977	OR659602
**14**	*Odorrana liboensis*	China: Guizhou: Libo: Maolan Nature Reserve	SYS a008240	PV347080	PV341295
**15**	*Odorrana liboensis*	China: Guangxi: Liuzhou City	SYS a008946	PV347084	PV341296
**16**	*Odorrana lipuensis*	China: Guangxi: Guilin City	SYS a004566	PV347087	PV341298
**17**	*Odorrana lipuensis*	China: Guangxi: Yangshuo County	SYS a004898	PV347088	PV341299
**18**	*Odorrana margaretae*	China: Sichuan: Emeishan: Mt. Emei	SYS a005303	ON391779	ON381770
**19**	*Odorrana schmackeri*	China: Hubei: Changyang: Gaojiayan Township	SYS a005476	OR658967	OR659619
**20**	*Odorrana tormota*	China: Anhui: Huangshan: Mt. Huangshan	SYS a002708	OR658945	OR659622
**21**	*Odorrana versabilis*	China: Guangxi: Longsheng: Huaping Nature Reserve	SYS a005117	OR658958	OR659624
**22**	*Odorrana wuchuanensis*	China: Guangdong: Yangshan: Jiangying Township	SYS a009109	PP388290	PV341304
**23**	*Odorrana yizhangensis*	China: Hunan: Yanling: Taoyuandong	SYS a001848	OR658941	OR659633
**24**	*Nidirana guangdongensis*	China: Guangdong: Yingde: Shimentai Nature Reserve	SYS a005767	MN946406	MN945162
**25**	*Nidirana nankunensis*	China: Guangdong: Longmen: Mt. Nankun	SYS a005719	MF807840	MF807879

**Table 2.** Measurements (in mm) of three adult female *Odorrana nagao* from Malipo, Yunnan, China.

**Character**	**SYS a009830**	**SYS a009831**	**SYS a009832**
**Sex**	Female	Female	Female
**SVL**	56.6	59.5	57.0
**HDL**	21.5	21.5	20.4
**HDW**	19.9	19.7	18.7
**SNT**	8.0	8.3	8.2
**IND**	5.6	5.9	5.5
**IOD**	5.4	5.4	4.9
**ED**	6.8	7.7	6.3
**TD**	4.4	4.8	4.1
**HND**	18.2	17.5	16.7
**RAD**	12.3	12.6	12.5
**FTL**	43.6	43.0	42.6
**TIB**	32.4	31.6	30.3

## References

[B14428929] AmphibiaChina The database of Chinese amphibians. http://www.amphibiachina.org/.

[B14428912] Chernomor O., Haeseler A., Minh B. Q. (2016). Terrace aware data structure for phylogenomic inference from supermatrices. Systematic Biology.

[B14428937] Clements R., Sodhi N. S., Schilthuizen M., Ng P. K.L. (2006). Limestone karsts of Southeast Asia: Imperiled arks of biodiversity. BioScience.

[B14428946] Fei L., Ye C. (2016). Amphibians of China.

[B14428962] Frost D. R. Amphibian Species of the World: an Online Reference. Version 6.2. https://amphibiansoftheworld.amnh.org/.

[B14428970] Hoang D. T., Chernomor O., Haeseler A., Minh B. Q., Vinh L. S. (2018). UFBoot2: Improving the ultrafast bootstrap approximation. Molecular Biology and Evolution.

[B14428980] Committee IUCN Standards and Petitions (2024). Guidelines for Using the IUCN Red List Categories and Criteria. Version 16. https://www.iucnredlist.org/resources/redlistguidelines.

[B14428988] Jiang K., Li J. T. (2021). Method for external measurement of adult anuran specimens. Bio-101.

[B14428997] Kalyaanamoorthy S., Minh B. Q., Wong T. K.F., Haeseler A., Jermiin L. S. (2017). ModelFinder: Fast model selection for accurate phylogenetic estimates. Nature Methods.

[B14429007] Katoh K., Kuma K. -I., Toh H., Miyata T. (2005). MAFFT version 5: Improvement in accuracy of multiple sequence alignment. Nucleic Acids Research.

[B14429016] Kimura M. (1980). A simple method for estimating evolutionary rates of base substitutions through comparative studies of nucleotide sequences. Journal of Molecular Evolution.

[B14429036] Lin S. S., Li Y. H., Su H. L., Yi H., Pan Z., Sun Y. J., Zeng Z. C., Wang J. (2022). Discovery of a new limestone karst-restricted odorous frog from northern Guangdong, China (Anura, Ranidae, *Odorrana*). ZooKeys.

[B14429025] Li S., Mu L., Jing J., Liu J., Cheng Y., Wang B. (2025). A new species of the genus *Odorrana* (Amphibia, Anura, Ranidae) from Guizhou Province, China. Zoosystematics and Evolution.

[B14508331] Liu Shuo, Zhao Junjun, Hou Mian, Rao Dingqi, Li Song (2026). First record of *Odorrana
nagao* Pham, Hoang, Nguyen, Nguyen, Ngo, Le & Ziegler, 2026 (Anura, Ranidae) from China. Check List.

[B14429059] Lyu Z. T., Zeng Z. C., Wan H., Yang J. H., Li Y. L., Pang H., Wang Y. Y. (2019). A new species of *Amolops* (Anura: Ranidae) from China, with taxonomic comments on *A.
liangshanensis* and Chinese populations of *A.
marmoratus*. Zootaxa.

[B14429071] Mo Y., Chen W., Wu H., Zhang W., Zhou S. (2015). A new species of *Odorrana* inhabiting complete darkness in a karst cave in Guangxi, China. Asian Herpetological Research.

[B14429125] Nguyen T. V., Vogel G. (2025). On the taxonomy of *Sibynophis
grahami* (Boulenger, 1904), with remarks on morphology, distribution, natural history, and conservation status (Squamata: Sibynophiidae: *Sibynophis*). Zootaxa.

[B14429112] Nguyen T. V., Li M. L., Huang J. J., Han B. Y., Nguyen D. T., Poyarkov N. A., David P., Ren J. L. (2025). Taxonomic reassessment of *Hebius
optatus* (Hu & Zhao, 1966) (Squamata: Natricidae), with the description of a new species from Vietnam and China. Asian Herpetological Research.

[B14429081] Nguyen T. V., Dubrovskaya A. S., Keetapithchayakul T. S., Le S. X., Eguchi K., Poyarkov N. A. (2026). Molecular and morphological evidence for considering *Odorrana
gigatympana* (Orlov, Ananjeva & Ho, 2006) a junior synonym of *Odorrana
absita*. Zootaxa.

[B14429101] Nguyen T. V., Dubrovskaya A. S., Keetapithchayakul T. S., Le S. X., Eguchi K., Poyarkov N. A. (2026). Rediscovery and taxonomic reassessment of *Rana
montivaga* Smith, 1921 (Anura: Ranidae), with transfer to the genus *Odorrana* Fei, Ye & Huang, 1990, based on morphology and molecular data. Zootaxa.

[B14429155] Pham C. T., Nguyen T. Q., Bernardes M., Nguyen T. T., Ziegler T. (2016). First records of *Bufo
gargarizans* Cantor, 1842 and *Odorrana
lipuensis* Mo, Chen, Wu, Zhang & Zhou, 2015 (Anura: Bufonidae, Ranidae) from Vietnam. Russian Journal of Herpetology.

[B14429134] Pham C. T., Hoang C. V., Nguyen M. H.T., Nguyen T. Q., Ngo H. N., Le D. T., Ziegler T. (2026). A new species of Odorrana (Anura, Ranidae) from the limestone karst forest of northern Vietnam. ZooKeys.

[B14429178] Poyarkov N. A., Nguyen T. V., Popov E. S., Geissler P., Pawangkhanant P., Neang T., Suwannapoom C., Orlov N. L. (2021). Recent progress in taxonomic studies, biogeographic analysis, and revised checklist of amphibians in Indochina. Russian Journal of Herpetology.

[B14429191] Poyarkov N. A., Nguyen T. V., Popov E. S., Geissler P., Pawangkhanant P., Neang T., Suwannapoom C., Ananjeva N. B., Orlov N. L. (2023). Recent progress in taxonomic studies, biogeographic analysis and revised checklist of reptiles in Indochina. Russian Journal of Herpetology.

[B14429165] Poyarkov N. A., Nguyen T. V., Le S. X., Le D. X., Arkhipov D. V., Gorin V. A., Hernandez A., Dufresnes C. (2024). Multiple lines of evidence for a new cryptic species of *Tylototriton* (Amphibia, Salamandridae) from northern Vietnam. Alytes.

[B14429205] Ronquist F., Teslenko M., Mark P., Ayres D. L., Darling A., Höhna S., Larget B., Liu L., Suchard M. A., Huelsenbeck J. P. (2012). MrBayes 3.2: Efficient Bayesian phylogenetic inference and model choice across a large model space. Systematic Biology.

[B14429231] Song H. M., Zhang S. Y., Qi S., Lyu Z. T., Zeng Z. C., Zhu Y. H., Huang M. H., Luan F. C., Shu Z. F., Gong Y., Liu Z. F., Wang Y. Y. (2023). Redefinition of the *Odorrana
versabilis* group, with a new species from China (Anura, Ranidae, *Odorrana*). Asian Herpetological Research.

[B14429220] Song H. M., Qi S., Wang H. T., Gong Y., Liu Y., Wang Y. Y. (2025). Definition and taxonomic revision of the karst-associated *Odorrana
lipuensis* group (Anura, Ranidae), with a new species from Guangdong, China. Zoosystematics and Evolution.

[B14429248] Song H. M., Zhou D. Y., Qi S., Wang Y. Y. (2026). First country record of *Tylototriton
koliaensis* Poyarkov et al., 2024 (Caudata, Salamandridae) from Guangxi, China. Check List.

[B14429257] Tamura K., Stecher G., Kumar S. (2021). MEGA11: Molecular Evolutionary Genetics Analysis version 11. Molecular Biology and Evolution.

[B14429266] Vogel G., Liu S., David P., Nguyen T. V. (2024). Solving of the long-term question: Resurrection of *Dinodon
yunnanensis* Werner, 1922 (Squamata: Colubridae: Lycodon. Zootaxa.

[B14429275] Wong T. K.F., Ly-Trong N., Ren H., Demotte P., Baños H., Roger A. J., Susko E., Bielow C., Maio N., Goldman N., Hahn M. W., Reis M., Vinh L. S., Huttley G., Lanfear R., Minh B. Q. (2026). IQ-TREE 3: Phylogenomic inference software using complex evolutionary models. Molecular Biology and Evolution.

[B14429296] Xu Y., Liao C., Poyarkov N. A., Weng S., Deng J., Bragin A., Zhang T., Nguyen T. V., Peng L. (2025). First record of *Scincella
fansipanensis* Okabe, Motokawa, Koizumi, Nguyen, Nguyen & Bui, 2024 (Squamata, Scincidae) from China. Biodiversity Data Journal.

[B14429310] Zhou B. (1956). Anatomy of Frogs.

